# Supplementary material for: Harnessing the Potential of Walnut Leaves from Nerpio: Unveiling Extraction Techniques and Bioactivity Through Caenorhabditis elegans Studies
Source: Foods. 2025 Mar 19;14(6):1048. doi: 10.3390/foods14061048 (PMC11942337; doi:10.3390/foods14061048)
Supplement: Supplementary file 1 [file foods-14-01048-s001.zip › foods-3468553-supplementary.pdf]

Article

Harnessing the Potential of Walnut Leaves from Nerpio: Unveiling Extraction Techniques and Bioactivity Through *Caenorhabditis elegans* Studies

Amel Hamdi [1,2,*], Miguel Angel Córdoba-Rojano [1], Jose Manuel Monje-Moreno [2], Elisa Guillén-Izquierdo [1], Rocío Rodríguez-Arcos [1], Ana Jiménez-Araujo [1], Manuel Jesús Muñoz-Ruiz [2] and Rafael Guillén-Bejarano [1,*]

[1] Instituto de la Grasa, Consejo Superior de Investigaciones Científicas (CSIC), Pablo de Olavide University Campus, Building 46, Carretera de Utrera Km 1, 41013 Seville, Spain; miguelcordobarojano@gmail.com (M.A.C.-R.); eligui99@hotmail.com (E.G.-I.); rrodri@ig.csic.es (R.R.-A.); araujo@ig.csic.es (A.J.-A.)

[2] Molecular Biology and Biochemical Engineering Department, Centro Andaluz de Biología del Desarrollo (CABD), University Pablo de Olavide (UPO), CSIC/UPO/JA, Carretera de Utrera Km 1, 41013 Seville, Spain; jmmonmor@upo.es (J.M.M.-M.); mmunrui@upo.es (M.J.M.-R.)

* Correspondence: amelhamdi1988@yahoo.fr (A.H.); rguillen@ig.csic.es (R.G.-B.)

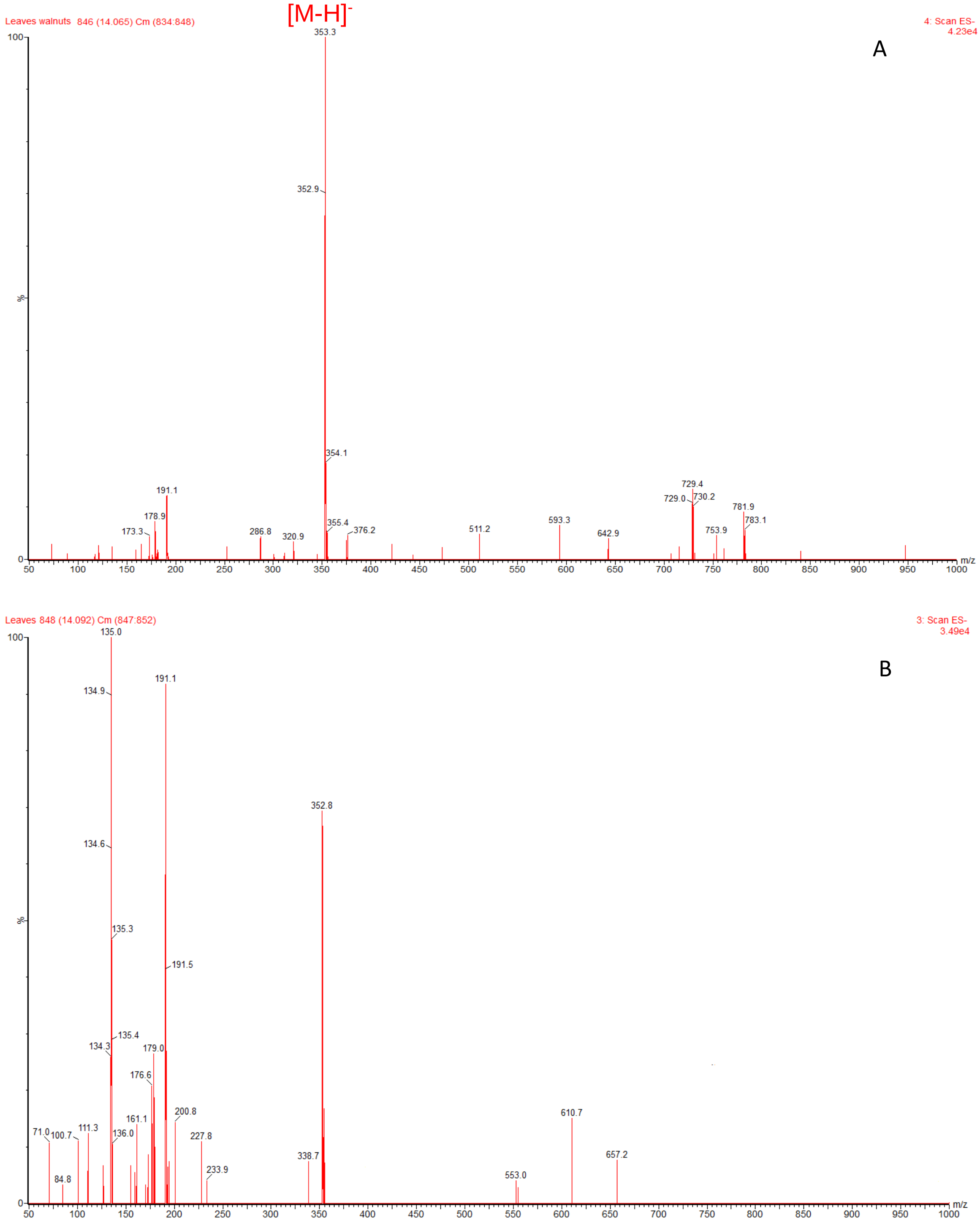

Figure S1. ESI spectra of *cis*-3-caffeoylquinic acid in negative mode at ionization energies of 50 (A) and 20 (B) eV

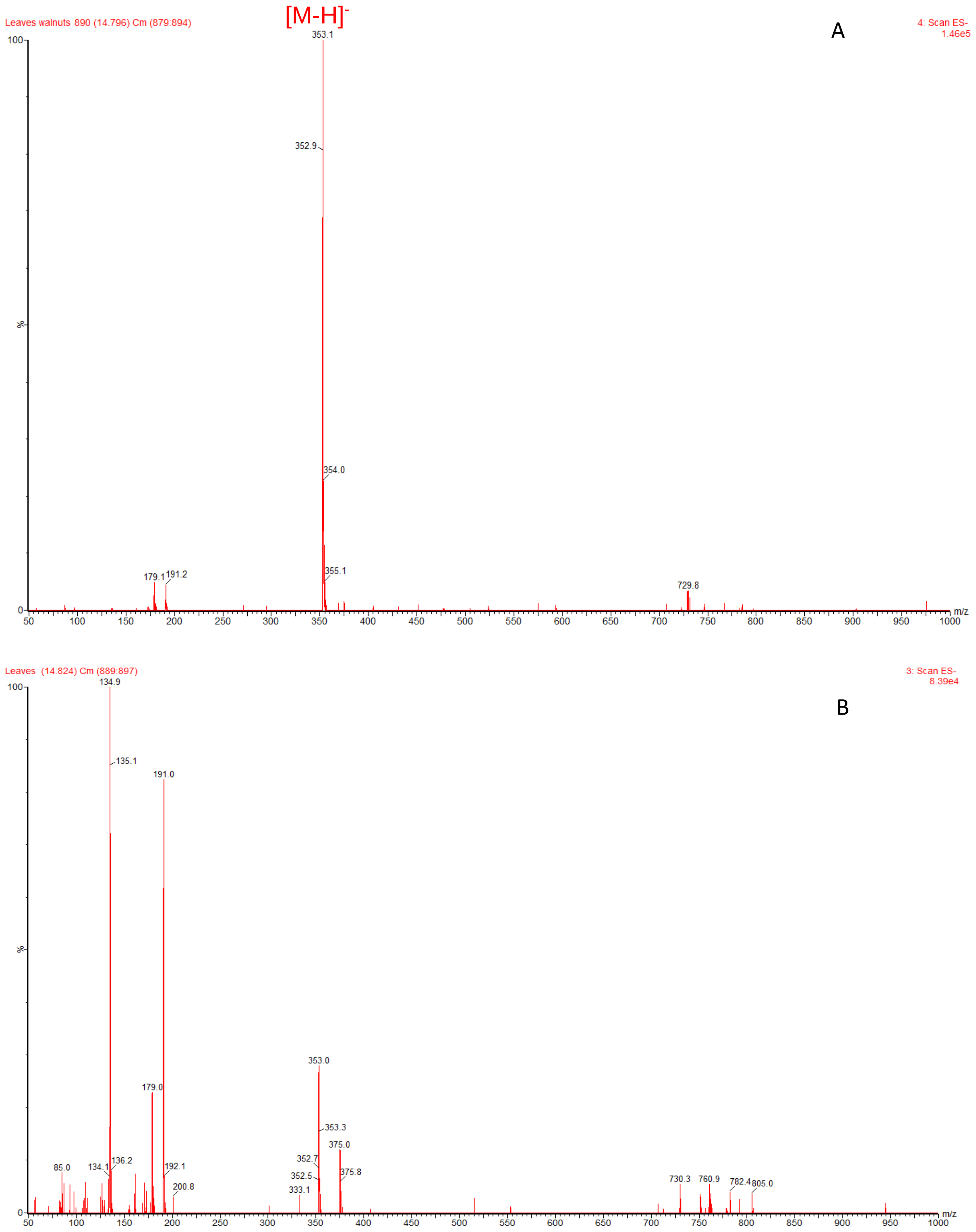

Figure S2. ESI spectra of *trans*-3-caffeoylquinic acid in negative mode at ionization energies of 50 (A) and 20 (B) eV

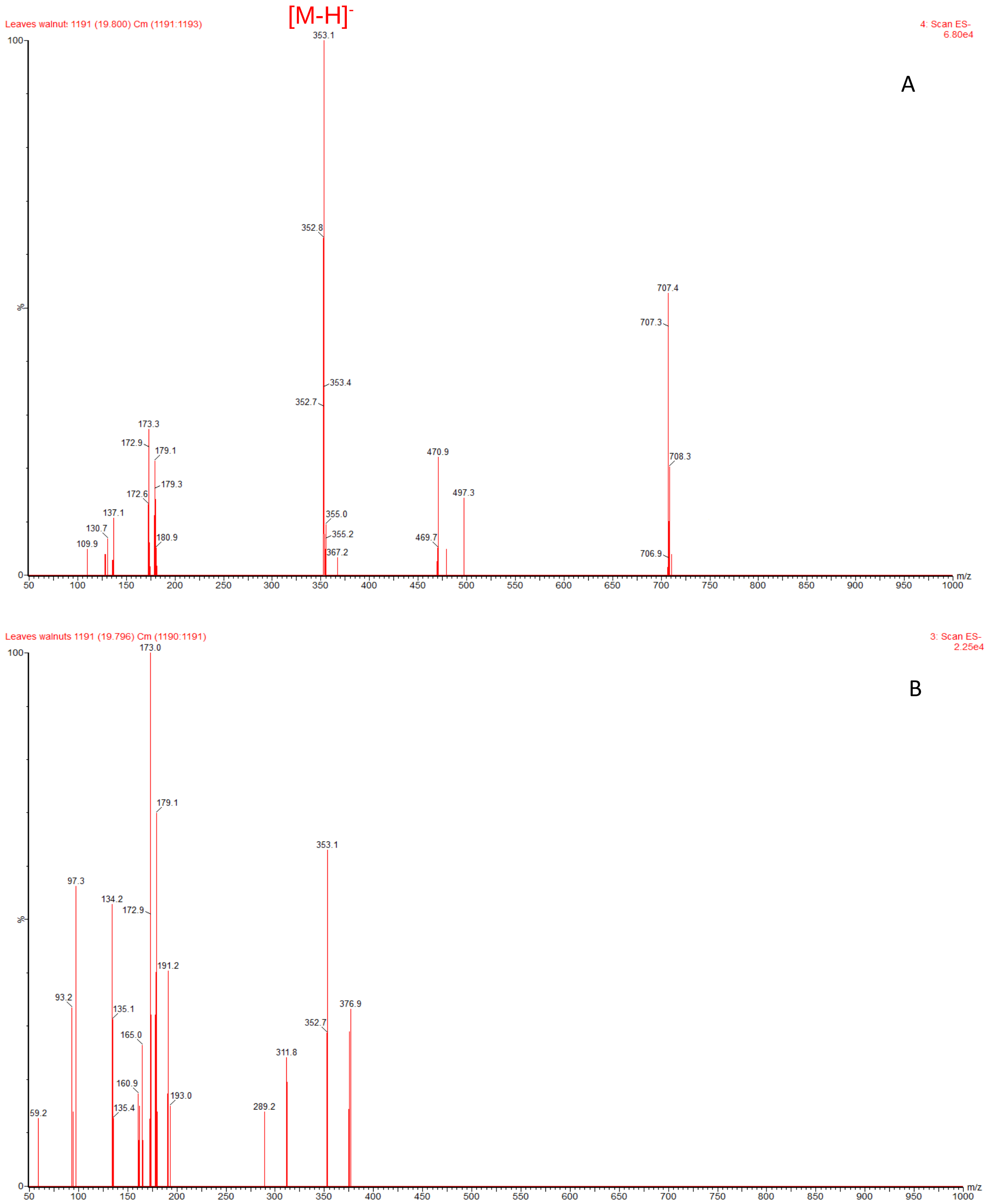

Figure S3. ESI spectra of 4-caffeoylquinic acid in negative mode at ionization energies of 50 (A) and 20 (B) eV

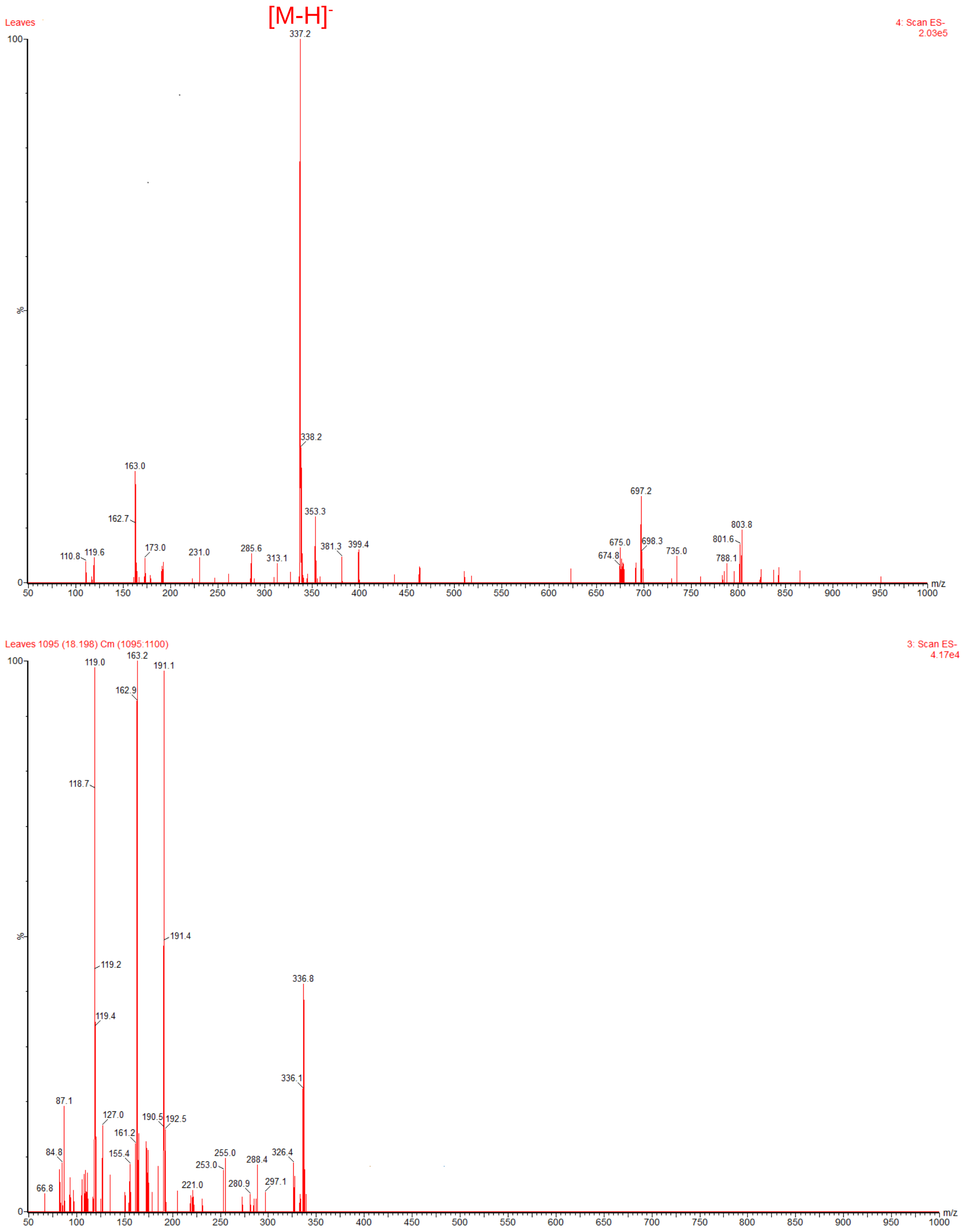

Figure S4. ESI spectra of *cis*-3-*p*-coumaroylquinic acid in negative mode at ionization energies of 50 (A) and 20 (B) eV

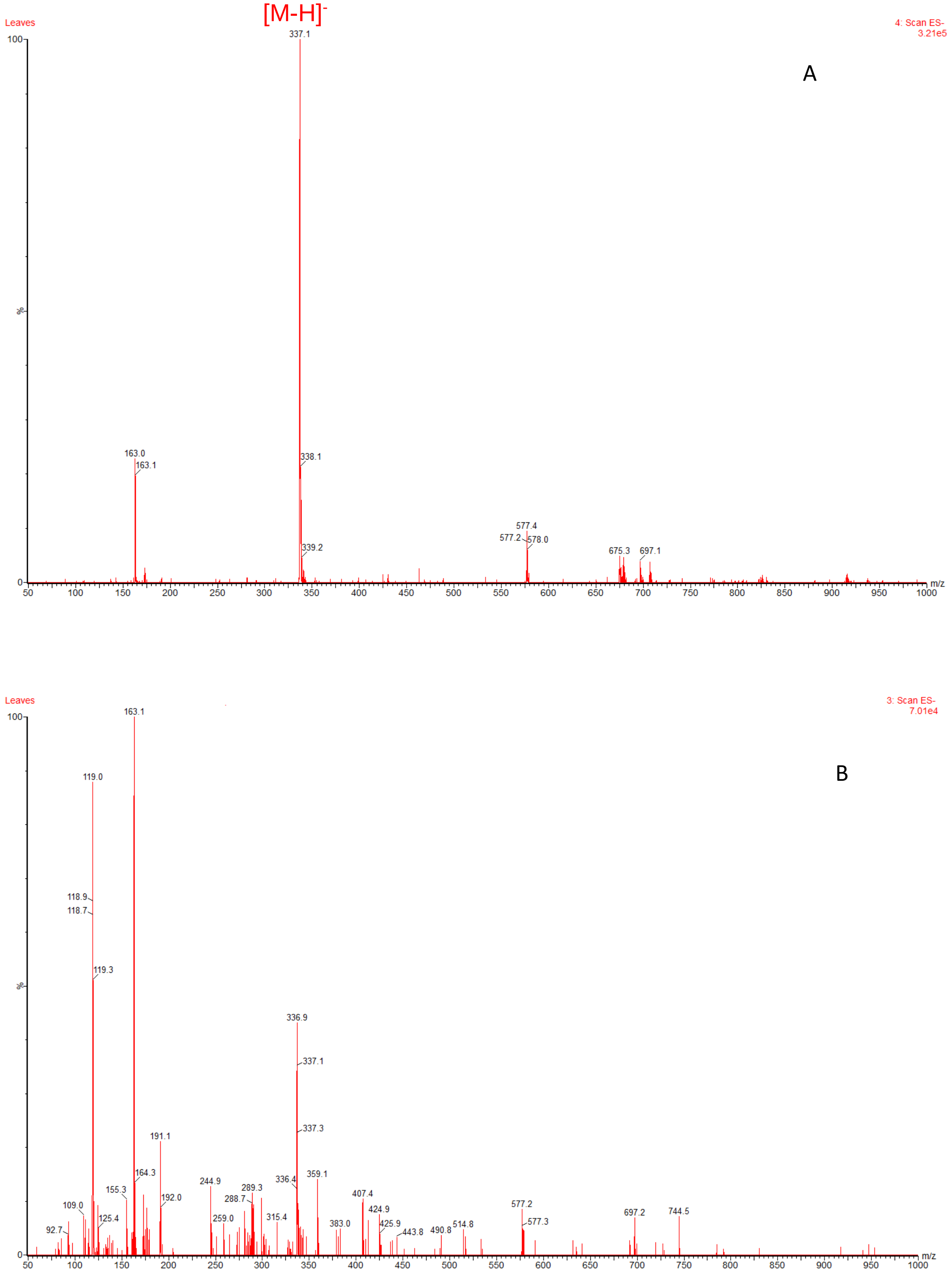

Figure S5. ESI spectra of *trans*-3-*p*-coumaroylquinic acid in negative mode at ionization energies of 50 (A) and 20 (B) eV

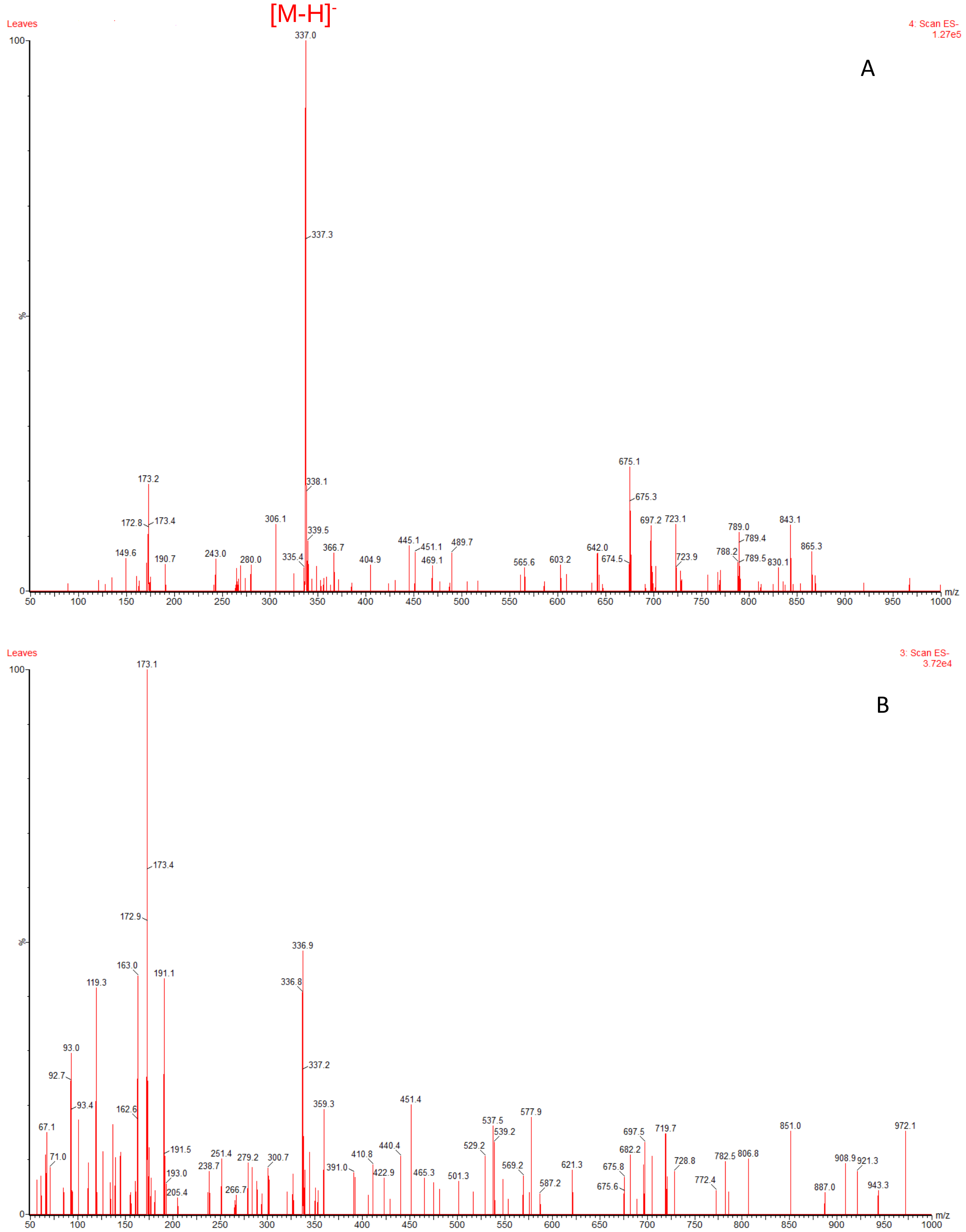

Figure S6. ESI spectra of *cis*-4-*p*-coumaroylquinic acid in negative mode at ionization energies of 50 (A) and 20 (B) eV

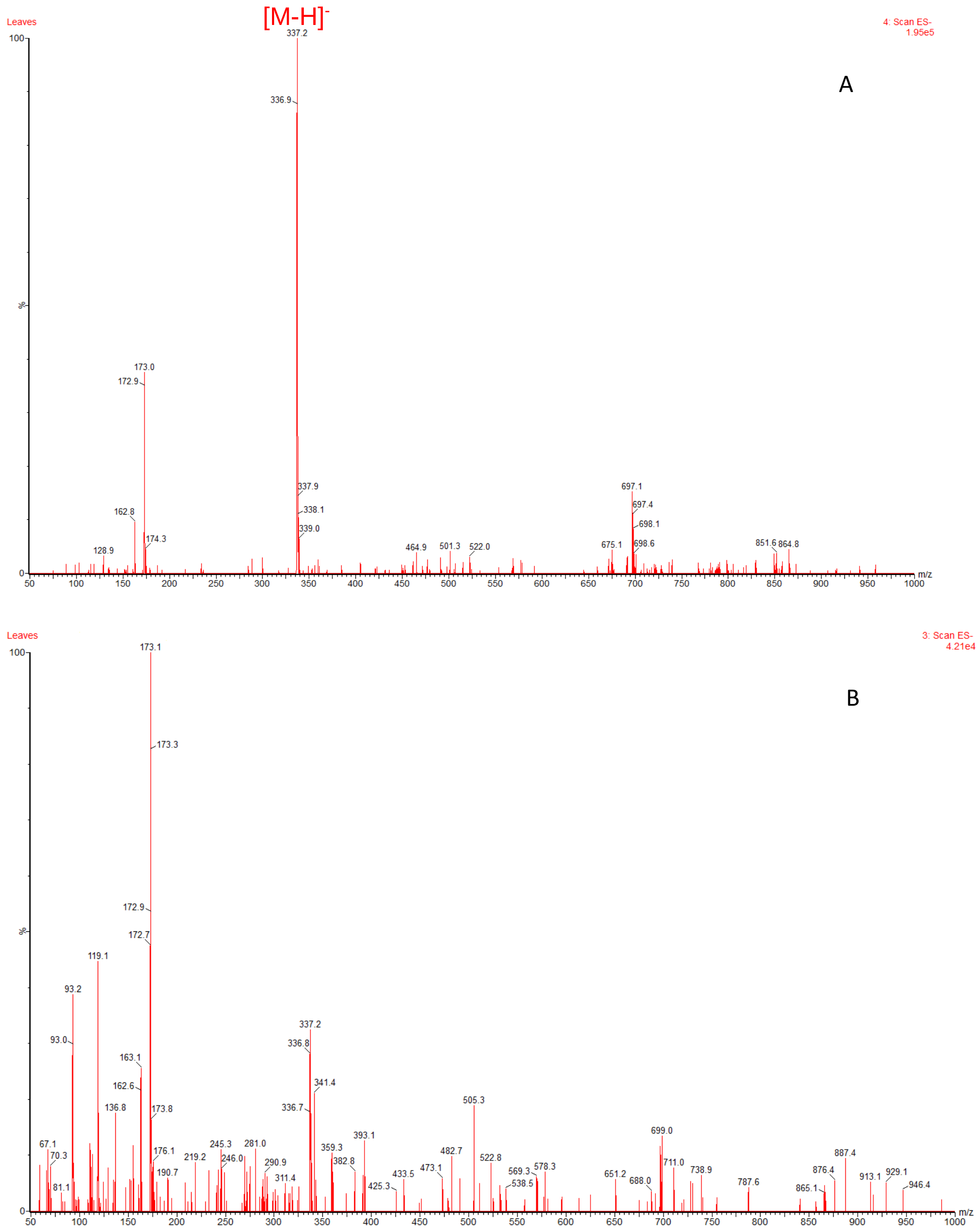

Figure S7. ESI spectra of *trans*-4-*p*-coumaroylquinic acid in negative mode at ionization energies of 50 (A) and 20 (B) eV

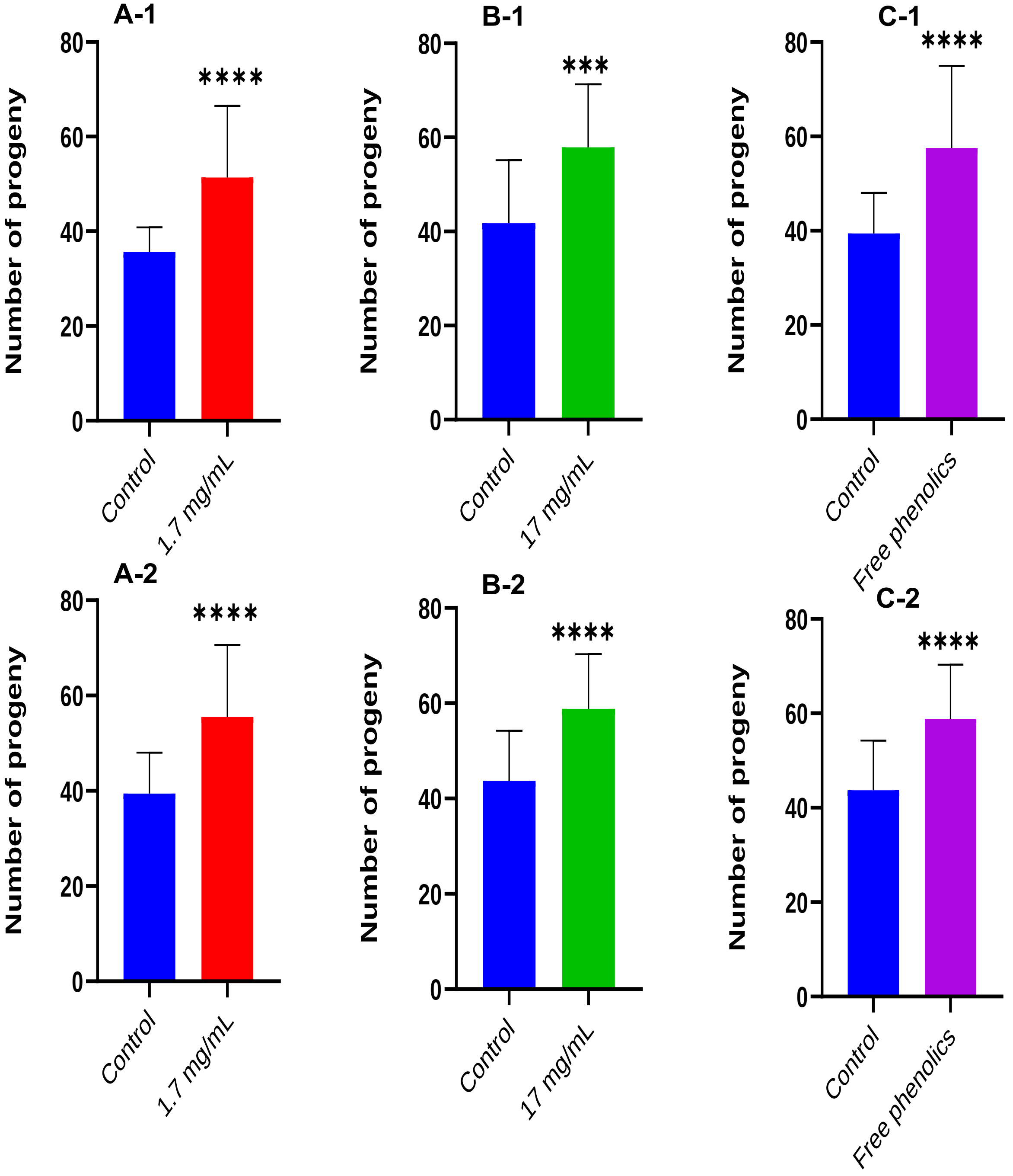

Figure S8. Progeny of *daf-2(e1370)* strain in the presence or absence (control) of walnut leaves extract at a concentration 1.7 and 17 mg/mL and in presence of free phenolics (A1/B1/C1 and A2/B2/C2, rep-resenting replicates 2 and 3, respectively). Data are presented as mean ± SD (n = 30 worms per group). Statistical significance (****$p < 0.001$, ***$p \leq 0.001$, **$p \leq 0.01$) was calculated using a two-tailed t-test compared to the control group.

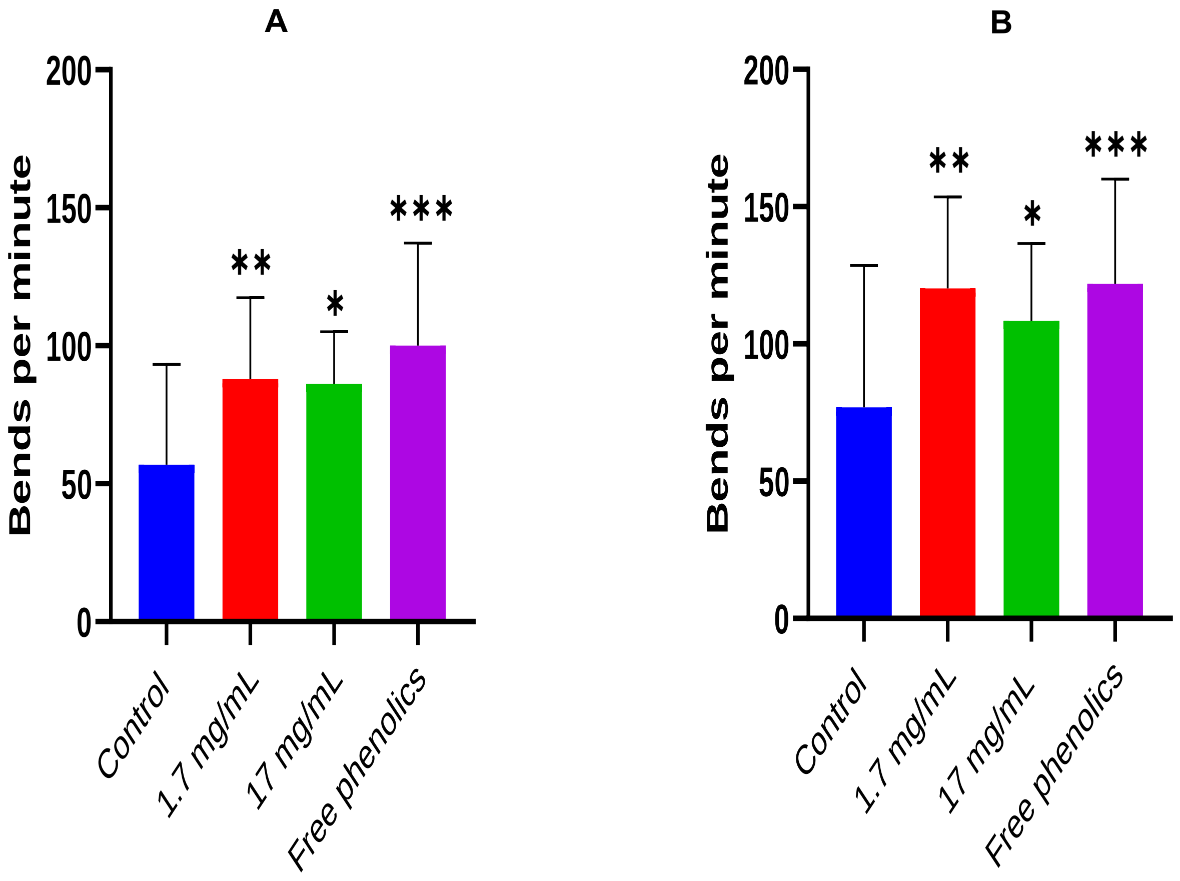

Figure S9. Effect of walnut leaf extract (1.7 and 17 mg/mL) and its free phenolics treatment on NL5901 worms' locomotion quantified as number of bends in one minute (n = 20/treatment) (A and B, representing replicates 2 and 3, respectively). Data are presented as mean ± SD (n = 20 worms per group). Statistical significance (*$p = 0.05$, **$p \leq 0.01$, ***$p \leq 0.001$) was determined by ANOVA with Dunnett's multiple comparisons test (vs. control).
